# Growth in Infancy and Childhood and Age at Menarche in Five Low- or Middle-Income Countries: Consortium of Health Orientated Research in Transitional Societies (COHORTS)

**DOI:** 10.1016/j.tjnut.2023.07.003

**Published:** 2023-07-13

**Authors:** Lukhanyo H. Nyati, Shane A. Norris, Lisa K. Micklesfield, Linda S. Adair, Caroline Fall, Nanette R. Lee, Reynaldo Martorell, Clive Osmond, Linda M. Richter, Harshpal S. Sachdev, Bernardo Horta, Aryeh D. Stein, COHORTS Group, Natalia P. Lima, Helen Goncalves, Bruna Goncalves C da Silva, Paula D. de Oliveira, Joseph Murray, Sara Naicker, Santosh K. Bhargava, Lakshmy Ramakrishnan, Sikha Sinha, Bhaskar Singh, Manuel Ramirez-Zea, Maria F. Kroker-Lobos, Isabelita Bas, Sonny Agustin Bechayda, Delia Carba, Tita Lorna Perez

**Affiliations:** 11 Pelotas Birth Cohorts; 12 Birth to Thirty; 13 New Delhi Birth Cohort; 14 INCAP Nutrition Supplementation Trial Longitudinal Study; 15 Cebu Longitudinal Health and Nutrition Survey; 1 SAMRC Developmental Pathways for Health Research Unit, Faculty of Health Sciences, University of the Witwatersrand, Johannesburg, South Africa; 2 Interprofessional Education Unit, Faculty of Community and Health Sciences, University of Western Cape, Cape Town, South Africa; 3 School of Human Development and Health and NIHR Southampton Biomedical Research Centre, University of Southampton, Southampton, United Kingdom; 4 Department of Nutrition, University of North Carolina, Chapel Hill, NC, United States; 5 MRC Lifecourse Epidemiology Centre, University of Southampton, Southampton, United Kingdom; 6 USC-Office of Population Studies Foundation, Inc., and Department of Anthropology, Sociology, and History, University of San Carlos, Cebu City, Philippines; 7 Hubert Department of Global Health, Rollins School of Public Health, Emory University, Atlanta, GA, United States; 8 DSI-NRF Centre of Excellence in Human Development, University of the Witwatersrand, Johannesburg, South Africa; 9 Sitaram Bhartia Institute of Science and Research, New Delhi, India; 10 Post-Graduate Program in Health and Behaviour, Universidade Católica de Pelotas, Pelotas, Brazil

**Keywords:** infant, linear growth, weight gain, obesity, age at menarche, birth cohort, low- and middle-income countries

## Abstract

**Background:**

Earlier age at menarche is associated with behavioral and noncommunicable disease risks. The influence of birth weight (BW) (intrauterine) and postnatal growth on age at menarche is not well studied in low- and middle-income countries (LMICs).

**Objective:**

Therefore, we investigated these associations in 5 LMIC birth cohorts.

**Methods:**

We analyzed data from Brazil, Guatemala, India, the Philippines, and South Africa (*n* = 3983). We derived stunting (< −2 SD scores) at 24 mo using the WHO child growth standards. We generated interaction terms with categorized BW and conditional weight (lighter < 0 or heavier ≥ 0), and height (shorter < 0 or taller ≥ 0) *z*-scores. We categorized early-, modal-, and late-onset menarche and used multilevel ordinal regression. We used multilevel linear regression on continuous age at menarche.

**Results:**

Mean age at menarche was 12.8 y (95% CI: 12.7 12.9). BW was not associated with age at menarche. Conditional height at 24 mo and mid-childhood (OR: 1.35; 95% CI: 1.27, 1.44 and 1.32; 1.25, 1.41, respectively) and conditional weight at 24 mo and mid-childhood (OR: 1.15; 1.08, 1.22 and 1.18; 1.11, 1.25, respectively) were associated with increased likelihood of early-onset menarche. Being heavier at birth and taller at 24 mo was associated with a 4-mo (95% CI: 0.8, 7.6) earlier age at menarche than being lighter at birth and shorter at 24 mo. Being heavier at birth but lighter in mid-childhood was associated with a 3-mo (95% CI: 0.8, 4.8) later age at menarche than being lighter at birth and mid-childhood. Age at menarche was 7 mo later in stunted than nonstunted girls.

**Conclusion:**

Age at menarche is inversely related to relative weight gain but also to rapid linear growth among those born shorter but remained stunted, and those born taller and grew excessively. These findings do not deter the global health goal to reduce growth faltering but emphasize the potential adverse effects of an obesogenic environment on adolescent development.

## Introduction

The secular trend of decreasing age at menarche in high-income countries has decelerated [1,2]. In North America and Europe, the age at menarche declined from 17 y in the mid-19th century to below 14 y by the mid-20th century [3,4]. Low- or middle-income countries (LMICs) are still experiencing a sharp decline in age at menarche [[5], [6], [7], [8]]. The mean age at menarche for South African Black females decreased from 14.9 y (95% CI 14.8, 15.0) in 1956 to 12.4 y (95% CI 12.2, 12.6) in 2006; an average decline of 0.50 y per decade [9], compared with approximately 0.08 y per decade among contemporary Dutch girls [2].

An earlier age at menarche is associated with lower attained height, increased adult obesity [[10], [11], [12], [13], [14]], some cancers [15], adult-onset diabetes [16], psychosocial disorders, and risky behaviors [17,18]. Therefore, it is useful to understand factors associated with age at menarche, as the potential life course implications for both health and socioeconomic outcomes for young girls and their offspring are profound.

Frisch et al. [19] first hypothesized that achieving some critical weight may be essential to trigger the onset of menarche and that the global rise in childhood weight over time may be the essential driver of the secular decline in menarche. However, there is some evidence that females who are lighter at birth may experience menarche at an earlier age than those born heavier and that the rate of weight gain during early childhood may be a critical determinant of the timing of puberty [20]. In Cebu, Philippines, girls who were relatively long (>49 cm) and light (<3 kg) at birth attained menarche ∼6 mo earlier than girls who were both short (<49 cm) and light (<3 kg) [21]. The association with low weight at birth was most pronounced among girls with greater than average linear growth in the first 6 mo of postnatal life [21]. In the UK MRC 1946 Birth Cohort, both linear growth and relative weight gain during infancy were negatively associated with age at menarche [22]. In the Dortmund Nutritional and Anthropometric Longitudinal Design study, both higher birth weight (BW) and faster postnatal weight gain to 24 mo were associated with earlier age at menarche, independent of prepubertal BMI [23].

Determining the relative contributions of BW, postnatal relative weight gain (considered separately from linear growth), and postnatal linear growth, to identify the age windows in which child growth is most strongly related to age at menarche is valuable to better understand predictors of puberty and potential drivers of pediatric obesity trajectories. Therefore, we aimed to investigate the independent associations of BW and postnatal linear growth and relative weight gain in infancy and childhood with age at menarche in 5 birth cohorts in LMICs.

## Methods

### Study populations

We analyzed data from birth cohorts in 5 LMICs: The 1982 Pelotas Birth Cohort (Brazil) [24], the Institute of Nutrition of Central America and Panama Nutrition Trial Cohort (Guatemala) [25], the New Delhi Birth Cohort (India) [26], the Cebu Longitudinal Health and Nutrition Survey (Philippines) [27], and the Birth to Thirty Cohort (South Africa) [28]. All field activities were reviewed and approved by an appropriate ethics committee or Institutional Review Board and all participants (or their parents, as appropriate) provided informed consent for all measures reported.

### Demographic characteristics and birthweight

Maternal age at the time of delivery and schooling were assessed using a questionnaire. In South Africa BW was obtained from reliable birth records [29], whereas the research teams in Brazil, India, and Guatemala measured BW. In Cebu, BW was based on a combination of interviewer-measured data (60%) and hospital records. As birth length was not measured for the India and South Africa studies, we use BW in all analyses.

### Measured anthropometric variables

Stature and weight were measured using standard methods and timings specific to each study. In all studies except Guatemala, supine length was measured up to 24 mo and standing height thereafter. In Guatemala, supine length was measured through age 7 y; we converted these measures to standing heights by subtracting 1.0 cm from lengths in children aged 24 mo and older. For the current analysis, we identified common ages of childhood measurements across the 5 sites and weight and length at 24 mo and at mid-childhood (age 48 mo for Brazil, Guatemala, India, and South Africa, and 8 y for the Philippines).

### Computed growth variables

*z*-Scores were calculated using WHO 2006 standards [30,31]. We considered girls to be stunted if their height-for-age *z*-score (HAZ) was < −2.0. Disentangling the consequences of linear growth and weight gain at different ages requires statistical methods to address the high correlation of weight with length and repeated measurements in the same individual over time. We used conditional size measures to eliminate these correlations. Conditional size measures are standardized residuals derived from regressing current size (represented as Fisher–Yates transformed weight or length/height *z*-scores) on all prior size measures [[32], [33], [34]]. Conditional variables represent children’s deviation from expected size based on their own prior measures and the growth of the other children in their cohort and can be interpreted as representing faster or slower relative weight gain or linear growth, respectively. For this analysis, the conditionals were generated for height-for-age and weight-for-age at 24 mo and mid-childhood, all represented as *z*-scores. The conditional variables were created separately for each site from the maximum sample with complete anthropometric data at all selected ages.

### Age at menarche

For Brazil, data on age at menarche were collected at age 15 y, with further updates in early adulthood for those that not yet had menses by 15 y. In Guatemala, the age at menarche was asked in adulthood (15–30 y). In India, the age at menarche was obtained at the age of 30 y. In the Philippines, age at menarche (month and year) was obtained by recall at several surveys (age 11, 15, 19, and 22 y) spanning adolescence through early adulthood. For the final age at menarche variable, the estimate obtained closest to the age at onset was used. In South Africa, age at menarche (month and year) was asked annually from age 9 y until 18 y and first prospectively reported age was used.

We treated age at menarche as both a continuous and categorical variable. Categorization of age at menarche as early, modal, or late was site-specific, based on modal age at menarche, expressed in integer years.

### Sample selection and statistical analyses

**FIGURE 1:**
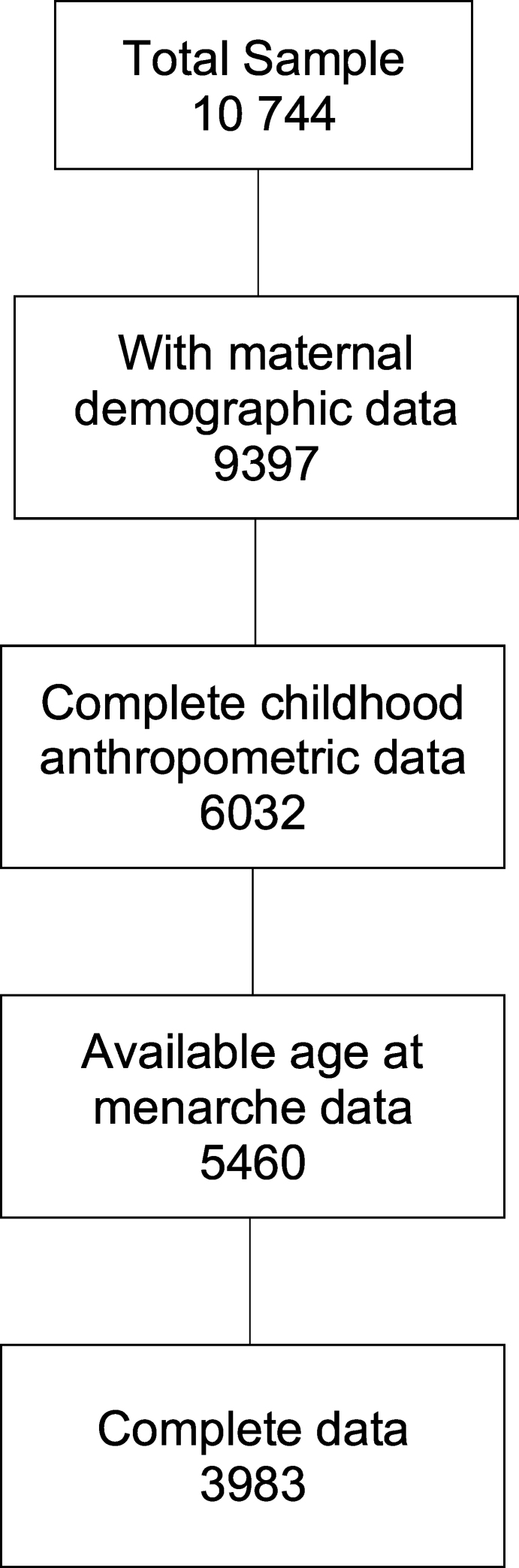
Flow diagram to show available data for key variables to formulate the analytical data set.

The total sample consisted of 10,774 female participants (Brazil 27%, Guatemala 11%, India 33%, South Africa 13%, and the Philippines 16%) with BW and maternal demographic characteristics. Complete anthropometric data were available for 6032 girls, of whom age at menarche was available for 3983 participants, who represent the sample for analysis (Figure 1). We performed sensitivity analyses by comparing participants excluded with those included in the analytical sample.

We characterized the individual cohorts using means and standard deviations (SDs) or categorical distributions, as appropriate. We pooled the data using mixed-effects (fixed and random effects) modeling with random slopes and intercepts for site. To assess risk of being classified as the early- or late-onset menarche group, we used mixed-effects ordinal regression. We performed a likelihood ratio test to assess proportionality of odds, and there was no difference in the relationship between the pairs of the levels of the ordinal outcomes. Furthermore, we used multilevel linear regression with maximum likelihood estimation to investigate the association of BW, and conditional weight (CW), and conditional height (CH) *z*-scores at 24 mo and in mid-childhood (CW24, CW48, CH24, and CH48) with age at menarche, with interactions between birthweight and the conditional measures. We plotted the residuals to assess homogeneity of variances, and a uniform pattern was observed after removing outliers. To disentangle the effects of birth size and postnatal growth, we dichotomized BW *z*-score below and equal to or above the site-specific median and CH and CW at 24 and 48 mo as <0 or ≥0, resulting in 4r levels for each interaction term, light-slow, light-fast, heavy-slow, and heavy-fast. We adjusted for maternal education (EDUC) as a proxy for the household socioeconomic environment, as it has been shown repeatedly to be associated with both early growth and age at menarche [21]. We also included the wealth index in our models, and the associations did not differ. We included the main effects and interaction terms in the same fixed effects model as follows:

(1) *Y*_ij_ = *β*_0_ + *β*_1i_∗BW_ij_ + *β*_2i_∗CW24_ij_ + *β*_3i_∗CW48_ij_ + *β*_4i_∗CH24_ij_ + *β*_5i_∗CH48_ij_ + *β*_6i_∗BW#CH24_ij_ + *β*_7i_∗BW#CH48_ij_ + *β*_8i_∗BW#CH24_ij_ + *β*_9i_∗BW#CH48_ij_ + *β*_10i_∗EDUC _ij_ + *ε*_ij_

where:

*Y*_ij_ is age at menarche for individual i in cluster j.

In the subsample with birth length, we used the dichotomized CH at 24 mo and stunting at 24 mo to generate 4 groups, slow-stunted, fast-stunted, slow-long, and fast-long. We used the 1-factor ANOVA with the assumption for equal variances to compare age at menarche between the 4 groups among children born short or long (HAZ <0 or ≥0). Finally, to assess differences in age at menarche between girls who were stunted and nonstunted at age 24 mo, we used a 2-sample *t*-test. All analyses were done using Stata software, version 13 (Stata Corporation, 2013), and we inferred significance at *P* < 0.05.

## Results

Characteristics of study participants are presented in Table 1. South African female participants were born to mothers with the highest number of years of schooling, compared with the other 4 cohorts. The South African participants were generally heavier and taller than their counterparts in Brazil, Guatemala, India, and Philippines. Stunting at 24 mo was most prevalent in Guatemala (77.3%) and least prevalent in Brazil (10.7%). Age at menarche differed by cohort, with the mean (SD) ranging from 12.4 (±1.4) y in Brazil to 13.7 (±1.2) y in India (Table 1).

**TABLE 1:** Selected characteristics of female participants in 5 birth cohorts (mean (SD) or %)

Variable	Brazil *n =* 1750 (44%)	Guatemala *n =* 154 (4%)	India *n =* 487 (12%)	Philippines *n =* 936 (24%)	South Africa *n =* 656 (16%)
Maternal age (y)	26.4 (6.3)	28.0 (6.9)	26.8 (5.6)	26.2 (6.0)	25.5 (6.3)
Maternal education (y)	6.6 (4.3)	1.2 (1.5)	5.6 (4.5)	6.9 (3.1)	9.7 (2.7)
Maternal height (cm)	156.4 (5.9)	148.7 (5.1)	152.0 (5.1)	150.5 (5.0)	158.9 (6.0)
Birthweight (kg)	3.2 (0.5)	3.0 (0.4)	2.8 (0.4)	3.0 (0.4)	3.0 (0.5)
Birthweight (*z*-score)	−0.2 (1.2)	−0.5 (1.0)	−1.1 (0.9)	−0.6 (1.0)	−0.5 (1.2)
Weight at 24 mo (kg)	10.8 (1.6)	9.6 (1.1)	9.7 (1.2)	9.5 (1.1)	11.2 (1.4)
Weight at 24 mo (*z*-score)	0.1 (1.0)	−1.6 (1.0)	−1.4 (1.01)	−1.7 (1.0)	−0.3 (1.0)
Weight at mid-childhood (kg)	15.3 (2.4)	13.4 (1.4)	13.2 (1.5)	20.33 (2.8)	15.2 (2.1)
Weight at mid-childhood (*z*-score)	−0.0 (1.0)	−1.4 (0.8)	−1.5 (0.9)	−1.8 (0.9)	−0.5 (0.9)
Birth length (cm)	—	48.8 (2.1)	48.1 (1.9)	48.9 (2.0)	-
Birth length (*z*-score)	—	−0.2 (1.15)	−0.5 (1.0)	−0.1 (1.1)	−
Height at 24 mo (cm)	80.2 (4.9)	76.9 (3.6)	79.5 (3.6)	78.4 (3.5)	82.8 (3.4)
Height at 24 mo (*z*-score)	−0.6 (1.2)	−2.8 (1.1)	−1.9 (1.1)	−2.3 (1.1)	−1.1 (1.04)
Stunted at 24 mo, %	10.7	77.3	45.0	59.6	17.7
Height at mid-childhood (cm)	97.0 (5.1)	92.6 (4.0)	93.8 (4.3)	117.5 (5.5)	98.9 (4.0)
Height at mid-childhood (*z*-score)	−0.7 (1.09)	−2.4 (0.9)	−2.1 (1.0)	−2.0 (0.9)	−0.9 (0.9)
Age at menarche (y)	12.4 (1.4)	13.5 (1.3)	13.7 (1.2)	13.1 (1.1)	12.7 (1.2)
Menarche					
Early onset (< modal age; %)	24.3	16.9	41.5	26.9	43.8
Modal onset (=modal age; %)	31.0	33.8	36.1	37.6	31.6
Later onset (>modal age; %)	44.7	49.4	22.4	35.5	24.7

Menarche categorization was site-specific, based on modal age at menarche: Brazil = 12 y, Guatemala = 13 y; Delhi = 14 y; Philippines = 13 y; South Africa = 13 y.

Mid-childhood was defined as 102 mo in the Philippines and 48 mo in all other countries.

Age at menarche, height at 24 mo, the prevalence of stunting at 24 mo, and height and weight in mid-childhood did not differ between participants excluded versus those included (Supplemental Table S1). For India, mothers of the girls who were included in the analysis had completed more schooling. Participants from Brazil who were included in the analyses were heavier at birth, whereas those included from Guatemala and the Philippines weighed slightly more at 24 mo. For Brazil and India, mothers of the girls who were included were slightly older, whereas those from South Africa were slightly younger.

In the ordinal regression analyses, the association between BW and age at menarche was null (Table 2). Risk of being in the early onset versus being in the modal or late-onset group increased by 35% (95% CI: 27, 44), 32% (95% CI: 25, 44), 15% (95% CI: 8, 22), and 18% (95% CI: 11, 25) per 1 SD increase in conditional lengths at 24 mo and mid-childhood and CW at 24 mo and mid-childhood, respectively. Conversely, risk of being in the later onset versus being in the modal or early-onset group decreased by 26% (95% CI: 21, 30), 24% (95% CI: 20, 29), 13% (95% CI: 7, 18), and 15% (95% CI: 10, 20) per 1 SD increase in conditional length at 24 mo and mid-childhood and CW at 24 mo and mid-childhood, respectively. Consistent patterns were seen across cohorts (site-specific models are presented in Supplemental Table S3, which shows the adjusted model).

**TABLE 2:** Pooled analysis examining associations between birth weight; conditional weight gain independent of length, and linear length gain independent of weight *z*-scores and menarche groups (OR; [95% CI])

Outcome variable: age at menarche (y) (*N* = 3983)	Model 2^1^ Adjusted for site and maternal education
Early onset vs. other groups combined	OR (95% CI)
Birth weight (*z*-score)	1.03 (0.98, 1.09)
Conditional height 2 y (*z*-score)	1.35 (1.27, 1.44)∗∗∗
Conditional height mid childhood (*z*-score)	1.32 (1.25, 1.41)∗∗∗
Conditional weight 2 y (*z*-score)	1.15 (1.08, 1.22)∗∗∗
Conditional weight mid childhood (*z*-score)	1.18 (1.11, 1.25)∗∗∗
Later onset vs. other groups combined	
Birth weight (*z*-score)	0.97 (0.92, 1.02)
Conditional height 2 y (*z*-score)	0.74 (0.70, 0.79)∗∗∗
Conditional height mid-childhood (*z*-score)	0.76 (0.71, 0.80)∗∗∗
Conditional weight 2 y (*z*-score)	0.87 (0.82, 0.93)∗∗∗
Conditional weight mid childhood (*z*-score)	0.85 (0.80, 0.90)∗∗∗

Early onset: age at menarche < modal age. Normal onset: age at menarche = modal age. Late onset: age at menarche > modal age.

Mid-childhood was defined as 102 mo in the Philippines and 48 mo in all other countries.

∗*P <* 0.05; ∗∗*P <* 0.01; ∗∗∗*P <* 0.001. Assessed using a multilevel ordinal logistic regression.

1 Coefficients for unadjusted models were similar to the adjusted models.

In both the unadjusted and adjusted multilevel linear regression models, 1 SD increase in conditional length at 2 y or in mid-childhood was associated with an ∼2-mo (95% CI: 1.2, 2.9) decrease in age at menarche (Table 3). For 1 SD increase in CW at 24 mo and in mid-childhood, there were decreases in age at menarche by ∼4 wk (95% CI: 1.0, 7.2). In the interaction results (Table 4), being heavy at birth and having faster linear growth between birth and age 24 mo was associated with ∼4-mo (95% CI: 0.8, 7.6) earlier age at menarche than girls who were light at birth and had slower linear growth. Conversely, being heavy at birth and experiencing slower weight gain between 24 mo and mid-childhood delayed age at menarche by ∼3 mo compared with girls who were born light and had slower weight gain between 24 mo and mid-childhood. For site-specific regression results, see Supplemental Figure S1A, B.

**TABLE 3:** Pooled analysis examining associations of birth weight and postnatal length and weight changes with age at menarche (y); (*β*-coefficients [95% CI])

Outcome variable: age at menarche (y) (*N =* 3983)	Model 2^1^ Adjusted for maternal education *β* (95% CI)
Birthweight (*z*-score)	−0.01 (−0.06, 0.04)
Conditional height at 24 mo (*z*-score)	−0.17 (−0.24, −0.10)∗∗∗
Conditional height at mid-childhood (*z*-score)	−0.17 (−0.23, −0.11)∗∗∗
Conditional relative weight at 24 mo (*z*-score)	−0.09 (−0.15, −0.03)∗∗
Conditional relative weight at mid-childhood (*z*-score)	−0.09 (−0.15, −0.02)∗∗

Mid-childhood was defined as 102 mo in the Philippines and 48 mo in all other countries.

∗*P <* 0.05; ∗∗*P <* 0.01; ∗∗∗*P <* 0.001. Assessed using a multilevel linear regression.

1 Coefficients for unadjusted models were similar to the adjusted models.

**TABLE 4:** Pooled analysis examining associations between birth weight and conditional height categories (interactions) and age at menarche (y)

Outcome variable: menarche (*N* = 3983)	Model 2^1^ Adjusted for maternal schooling *β* (95% CI)
Birth weight and conditional height categories 24 mo	
Light-slow	Ref.
Light-fast	−0.09 (−0.23, 0.05)
Heavy-slow	−0.24 (−0.50, 0.02)
Heavy-fast	−0.35 (−0.63, −0.07)∗
Birth weight and conditional height categories in mid-childhoodv	
Light-slow	Ref.
Light-fast	−0.07 (−0.21, 0.06)
Heavy-slow	0.10 (−0.07, 0.26)
Birth weight and conditional weight categories 24 mo	
Light-slow	Ref.
Light-fast	0.08 (−0.06, 0.21)
Heavy-slow	0.09 (−0.07, 0.26)
Birth weight and conditional weight categories in mid-childhood	
Light-slow	Ref.
Light-fast	−0.08 (−0.21, 0.05)
Heavy-slow	0.24 (0.07, 0.40)∗∗

Mid-childhood was defined as 102 mo in the Philippines and 48 mo in all other countries.

∗ *P <* 0.05; ∗∗*P* < 0.01; ∗∗∗*P* < 0.001. Assessed using a multilevel linear regression.

1 Coefficients for unadjusted models were similar to the adjusted models.

In the subsample with birth length (Supplemental Table S2), being in the fast-stunted (13.1 ± SD, 1.3) group was associated with earlier age at menarche than being in the slow-stunted (14.0 ± 1.3) or slow-long groups (13.6 ± 1.1), among girls who were shorter at birth (*z*-score < 0). Additionally, being in the fast-long (13.3 ± 1.2) group was associated with earlier age at menarche than being in the slow-stunted group. Among girls who were taller at birth (*z*-score ≥ 0), being both in the fast-stunted (13.0 ± 1.2) and fast-long (12.9 ± 1.2) groups was associated with earlier age at menarche than being in the slow-stunted (13.6 ± 1.2) or slow-long (13.5 ± 1.1) groups.

Across all cohorts, girls stunted at 24 mo had a 7.2 mo (95% CI 7.0, 8.9) later mean age at menarche compared with girls not stunted (Table 5).

**TABLE 5:** Age at menarche (mean (SD)) among girls in 5 birth cohort studies in low- or middle-income countries, by stunting category at age 24 mo

Study site	Not stunted at 24 mo		Stunted at 24 mo	
	Mean (SD)	*N*	Mean (SD)	*N*
Brazil	12.3 (1.4)	1662	12.8 (1.7)∗∗∗	88
Guatemala	13.4 (1.4)	35	13.5 (1.3)	119
India	13.5 (1.2)	268	14.0 (1.2)∗∗∗	219
Philippines	12.9 (1.0)	378	13.3 (1.0)∗∗∗	558
South Africa	12.6 (1.2)	540	12.9 (1.2)∗	116
All sites	12.6 (1.4)	2883	13.3 (1.3)∗∗∗	1200

∗*P <* 0.05; ∗∗*P <* 0.01; ∗∗∗*P <* 0.001. Assessed using a *t*-test.

## Discussion

In this study, we assessed associations of BW and subsequent linear growth and weight gain with age at menarche in birth cohorts in 5 LMICs. The longitudinal data allowed us to examine growth and weight gains among infants and toddlers (birth to 2 y) and in mid-childhood and their association with onset and age at menarche. In the pooled analyses, BW was not associated with age at menarche, but greater linear growth and greater relative weight gain in both infancy and early childhood were each associated with a reduction in age at menarche, and hence a greater likelihood of having a relatively early menarche. The direction of the associations was consistent across countries, although not all estimates achieved significance. Girls who were stunted at the age of 2 y had a later age at menarche than girls who were not stunted. Extending previous research from the Cebu cohort, we found a synergistic interaction between BW and postnatal linear growth, such that girls who were both heavier at birth and were taller at 2 y had an earlier age at menarche compared with those born light who also experienced lower than average postnatal growth.

Frisch et al. [19] were the first to hypothesize a “direct relation between critical weight and menarche” and that this relationship accounts for the delay in the onset of menarche due to undernutrition, and why secular trends to earlier age at menarche are occurring in countries with improved nutrition. This has been supported by several earlier population studies, however, greater prepubertal height and BMI have also been shown to be associated with earlier menarche in high-income country populations [35]. Leptin, insulin, and other hormones may accelerate pubertal onset and these biomarker concentrations are considerably higher in overweight or obese girls. ^[^^36^^]^ Our work extends previous research in high-income countries to the LMIC context and highlights not only that absolute weight or height are important predictors of age at menarche, but that both greater weight gain and linear growth in infancy and childhood contribute to an earlier onset of menarche. Exploratory analysis in the subsample with birth length showed that the earliest age at menarche was among girls who were born shorter, grew faster but remained stunted at 24 mo, and those who were born taller and experienced faster growth, potentially crossing the centile lines. These findings suggest that the efforts to reduce the burden of stunting do not simultaneously increase the prevalence of overweight and obesity and are unlikely to further contribute to the declining age at menarche.

Interestingly, BW was not associated with age at menarche in this study, contrary to some findings from high-income countries (HICs). However, from previous Cebu cohort results, while BW was not a predictor, body proportion was; indicating that it is important to not discount a potential prenatal effect [21]. Furthermore, Adair [21] concluded that there is an important maturational trajectory established prenatally, but also affected by postnatal growth. In our analyses, girls who were heavier at birth and had faster linear growth (but not larger relative weight gain) experienced a lower age at onset of menarche compared with girls who were lighter at birth and had slower linear growth between birth and 2 y. Additionally, we showed that being heavy at birth and having slower weight gain between 2 y and mid-childhood was associated with a delay in age at menarche.

In LMICs, where child malnutrition is widespread and height is linked to educational, health, and economic outcomes [37] and with offspring health [38], early menarche may be an important intermediate factor. Studies in Europe have shown that constrained prenatal growth may accelerate progression to menarche and shortens growth spurt durations, possibly resulting in shorter attained height [39]. A recent systematic review highlighted that an early onset of menarche was associated with early sexual debut, adolescent pregnancy, and some sexually transmitted infections in LIMCs [40]. In the South African cohort, both height and BMI in early childhood predicted the trajectory of pubertal development; furthermore, the progression through puberty modified the relation between prepubertal and adult body size suggesting that an early onset of puberty may place girls at a greater risk of developing overweight or obesity in adulthood [41,42].

Given that a large number of studies have been carried out in HICs, our study that used data from 5 LMICs offers a valuable contribution to the literature. We were able to obtain a large sample with common data points, which allowed us to characterize early childhood growth patterns in detail, assessing the independent and joint contributions of body size and growth. A limitation in the current analyses is that not all cohorts had access to data on length at birth, which precluded the assessment of relations between different birth length combinations and menarche. We also did not adjust for feeding patterns, both in childhood and adolescence. There was variability in age and consequently anthropometric measurements at the mid-childhood time point between the Philippines versus other cohorts. Although children in the Philippines were closer to puberty, none had reached puberty at this stage. Additionally, we converted the anthropometric data to conditional variables, which assessed the growth rate between 2 time points, making the data comparable. Although the findings were not weighted for differences in sample size between the sites, data were pooled using mixed-effects modeling, which handles imbalanced data well.

In conclusion, age at menarche is related to weight gain (as postulated many decades ago) but also to linear growth. Given the known associations between BW, early growth, and the adult outcomes of schooling, attained height, body composition, blood pressure, and impaired fasting glucose [34,[43], [44], [45], [46]], and the emerging evidence that puberty may be an important intermediate product of earlier growth and a mediator of adult outcomes, it is of critical importance to better understand the relation of pubertal timing to later adult health outcomes. Rapid linear growth, regardless of birthweight status was associated with an earlier age at menarche. Faster linear growth between birth and 2 y was associated with an earlier age at menarche among girls who were born shorter and remained stunted and those who were born taller but grew excessively. Slower weight gain between 2 and 4 y was associated with a delay. Thus, these findings do not deter the global health goal to reduce growth faltering but further emphasize the potential adverse effects of an obesogenic environment on adolescent behavioral risks and the potential future vulnerability of children who experience inadequate catch-up growth.

## Funding

The Wellcome Trust (089257/Z/09/Z) has funded the COHORTS collaboration. The Bill and Melinda Gates Foundation (OPP1020058 and OPP1164115) provided additional funding for data analysis for this paper. Funding for the individual cohorts was as follows: Pelotas Birth Cohort (Brazil): Wellcome Trust; INCAP Nutrition Trial Cohort Study (Guatemala): the US National Institutes of Health; New Delhi Birth Cohort Study (India): Indian Council of Medical Research, US National Center for Health Statistics, British Heart Foundation, Medical Research Council (UK) and the UK Department for International Development (DFID) under the MRC/DFID concordat; Cebu Longitudinal Health and Nutrition Study (the Philippines): US National Institutes of Health; Birth to Twenty (South Africa): Wellcome Trust, South African Medical Research Council and University of the Witwatersrand. S.A.N., L.K.M., and L.M.R. are supported by the DST-NRF Centre of Excellence in Human Development at the University of the Witwatersrand, Johannesburg, South Africa.

## Author contributions

The authors’ responsibilities were as follows – SAN, LHN, ADS: conceptualized and reviewed the analysis. SAN, LHN analyzed data and wrote the first draft manuscript and revisions. ADS edited the first draft and revisions. All authors read and approved the manuscript.

## Conflicts of Interest

The authors report no conflicts of interest.

## Data availability

Data will be available upon reasonable request to the principal investigators at each study site.

## Appendix A. Supplementary data

Multimedia component1 Multimedia component1
