# Supplementary material for: Growth in Infancy and Childhood and Age at Menarche in Five Low- or Middle-Income Countries: Consortium of Health Orientated Research in Transitional Societies (COHORTS)
Source: J Nutr. 2023 Jul 13;153(9):2736–43. doi: 10.1016/j.tjnut.2023.07.003 (PMC10517227; doi:10.1016/j.tjnut.2023.07.003)
Supplement: Multimedia component1 [file mmc1.docx]

**Supplementary table 1: Characteristics of included and excluded participants**

|  | Brazil | | Guatemala | | India | | Philippines | | South Africa | |
| --- | --- | --- | --- | --- | --- | --- | --- | --- | --- | --- |
|  | Included | Excluded | Included | Excluded | Included | Excluded | Included | Excluded | Included | Excluded |
| N (%) | 1750 (61) | 1126 (39) | 154 (13) | 1008 (87) | 487 (14) | 3119 (86) | 936 (65) | 512 (35) | 656 (39) | 1026 (61) |
| Maternal schooling (years) | 6.6 (4.3) | 6.5 (4.3) | 1.3 (1.5) | 1.2 (1.6) | 5.6 (4.5) | 5.1 (4.6) | 6.9 (3.1) | 7.4 (3.4) | 9.7 (2.7) | 9.4 (3.2) |
| Maternal age (years) | 26.4 (6.3) | 25.2 (6.0) | 28.0 (6.9) | 26.8 (7.3) | 26.8 (5.6) | 25.9 (5.1) | 26.2 (6.0) | 25.9 (5.8) | 25.5 (6.3) | 26.2 (5.9) |
| Birthweight (kg) | 3.2 (0.5) | 3.1 (0.6) | 3.0 (0.4) | 3.0 (0.5) | 2.8 (0.4) | 2.7 (0.4) | 3.0 (0.4) | 2.9 (0.5) | 3.0 (0.5) | 3.0 (0.5) |
| Weight at 24mo (kg) | 10.8 (1.6) | 10.8 (1.6) | 9.6 (1.1) | 9.3 (1.2) | 9.7 (1.2) | 9.8 (1.3) | 9.5 (1.1) | 9.3 (1.2) | 11.2 (1.4) | 11.2 (1.3) |
| Height at 24mo (cm) | 80.2 (4.9) | 80.2 (5.2) | 76.9 (3.6) | 76.4 (3.7) | 79.5 (3.6) | 79.6 (3.9) | 78.4 (3.5) | 78.1 (3.9) | 82.8 (3.4) | 83.3 (3.6) |
| Stunting (%) | 188 (10.7) | 64 (11.4) | 119 (77.3) | 291 (79.7) | 219 (45.0) | 945 (45.9) | 558 (59.6) | 160 (64.0) | 116 (17.7) | 43 (15.3) |
| Weight at 48mo (kg) | 15.3 (2.4) | 15.4 (2.4) | 13.4 (1.4) | 13.3 (1.5) | 13.2 (1.5) | 13.3 (1.7) | 20.3 (2.8) | 20.8 (3.7) | 15.2 (2.1) | 15.4 (1.9) |
| Height at 48mo (cm) | 97.0 (5.1) | 97.4 (5.3) | 92.6 (4.0) | 92.1 (4.4) | 93.8 (4.3) | 94.2 (4.7) | 117.6 (5.5) | 117.8 (6.1) | 98.9 (4.0) | 98.9 (4.3) |
| Age at menarche (years) | 12.4 (1.4) | 12.4 (1.4) | 13.5 (1.3) | 13.6 (1.4) | 13.7 (1.2) | 13.8 (1.2) | 13.1 (1.0) | 13.0 (0.9) | 12.7 (1.2) | 12.7 (1.2) |

**Supplementary Table 2: Differences in age at menarche between categories of growth rate (birth to 2y) and stunting classification (2y) among girls born shorter (SDS < 0) and those born taller (=>0).**

|  |  | Slow-stunted | Fast-stunted | Slow-long | Fast-long |
| --- | --- | --- | --- | --- | --- |
| Shorter at birth < 0 SDS | N | 24 | 291 | 488 | 126 |
|  | Mean (SD) | 14.0 (1.3)^1**;3*^ | 13.1 (1.2)^4***^ | 13.6 (1.1) | 13.3 (1.2) |
| Taller at birth => 0 SDS | N | 39 | 318 | 214 | 41 |
|  | Mean (SD) | 13.6 (1.2)^1*;3*^ | 13.0 (1.1)^4***^ | 13.5 (1.1)^6**^ | 12.9 (1.2) |
| * p<0.05; ** p< 0.01; ***p< 0.001. Assessed using an ANOVA assuming equality of variance  Differences: 1 - Slow-stunted vs. Fast-stunted; 2 - Slow-stunted vs. Slow-long; 3 - Slow-stunted vs. Fast-long; 4 - Fast-stunted vs. Slow-long;5 - Fast-stunted vs. Fast-long; 6 - Slow-long vs. Fast-long  SDS – standard deviation scores | | | | | |

**Supplementary Table 3: Site-specific associations between birthweight; conditional weight and height gain Z-scores and menarche groups among participants (adjusted for maternal education). Odds Ratio; OR (95% Confidence Intervals) are presented.**

| **Variable** | **Brazil**  **(N=1750)** | **Guatemala**  **(N=154)** | **India**  **(N=487)** | **Philippines**  **(N=936)** | **South Africa**  **(N=656)** |
| --- | --- | --- | --- | --- | --- |
| **Early onset vs. other groups combined** | OR (95% CI) | OR (95% CI) | OR (95% CI) | OR (95% CI) | OR (95% CI) |
| Birthweight (Z-score) | 1.02 (0.94 - 1.10) | 1.21 (0.87 - 1.67) | 1.06 (0.89 - 1.26) | 1.08 (0.95 - 1.23) | 1.01 (0.89 - 1.15) |
| Conditional height gain 0-2 years (Z-score) | 1.29 (1.17 - 1.42)*** | 1.24 (0.91 - 1.70) | 1.28 (1.06 - 1.56)* | 1.51 (1.32 - 1.72)*** | 1.37 (1.18 - 1.59)*** |
| Conditional height gain 2-4 years (Z-score) | 1.28 (1.16 - 1.40)*** | 1.21 (0.89 - 1.65) | 1.15 (0.97 - 1.37) | 1.72 (1.51 - 1.96)*** | 1.22 (1.06 - 1.41)** |
| Conditional weight gain 0-2 years (Z-score) | 1.16 (1.06 - 1.26)** | 1.34 (0.97 - 1.84) | 0.91 (0.76 - 1.09) | 1.16 (1.02 - 1.32)* | 1.23 (1.06 - 1.43)** |
| Conditional weight gain 2-4 years (Z-score) | 1.19 (1.09 - 1.31)*** | 0.87 (0.64 - 1.18) | 1.01 (0.86 - 1.20) | 1.50 (1.32 - 1.71)*** | 1.05 (0.91 - 1.21) |
| **Early onset**: Age at menarche < Modal age. **Normal onset**: Age at menarche = Modal age. **Late onset**: Age at menarche > Modal age  *p<0.05; **p< 0.01; ***p< 0.001. Assessed using ordinal logistic regression  All models adjusted for maternal education  ^φ^Coefficients for unadjusted models were similar to the adjusted models  Mid-childhood was defined as 102 months in the Philippines and 48 months in all other countries | | | | | |


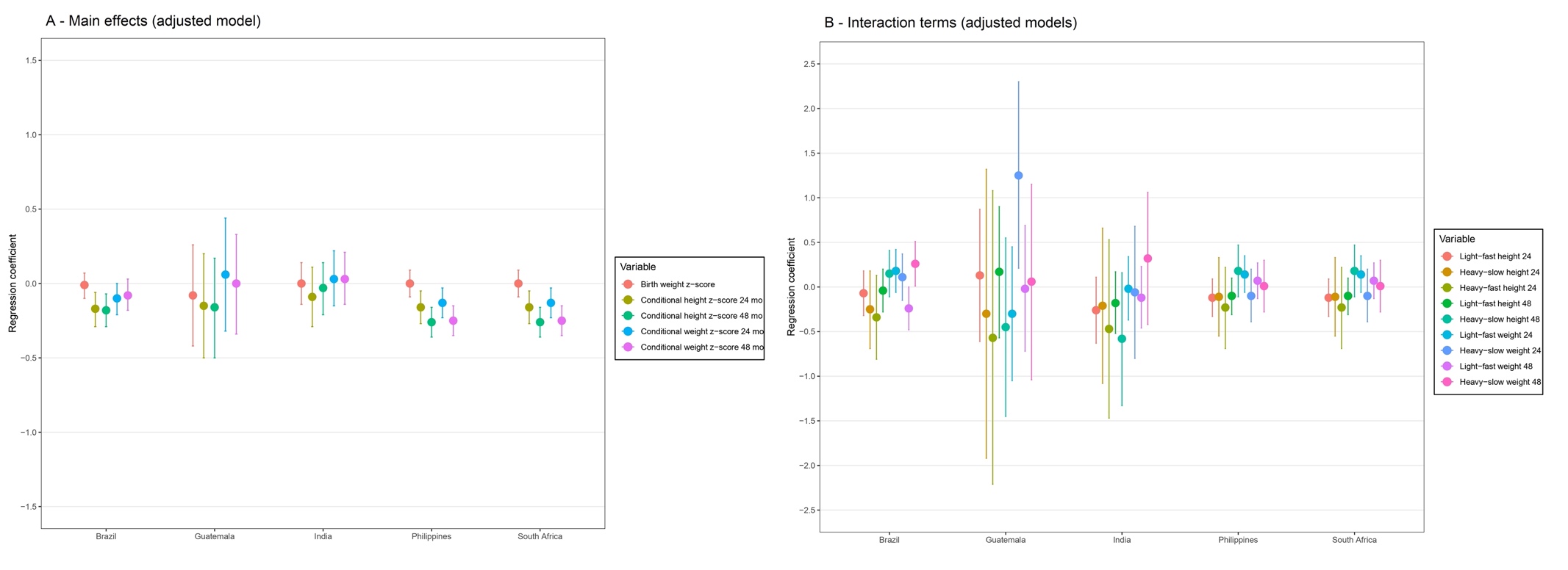


**Supplementary Figure 1: Site-specific associations between age at menarche and (a) the main effects (birthweight; conditional relative weight and linear length gain Z-scores), and (b) the categorised interaction terms (adjusted for maternal education). Coefficients ± 95% Confidence Intervals are presented.**
